# Supplementary material for: Electric Field-Induced Settling and Flotation of Flocs in Mixed Aqueous Suspensions of Poly(methyl methacrylate) and Aluminosilicate Hollow Particles
Source: Materials (Basel). 2025 Mar 14;18(6):1289. doi: 10.3390/ma18061289 (PMC11943974; doi:10.3390/ma18061289)
Supplement: Supplementary file 1 [file materials-18-01289-s001.zip › materials-3521145-supplementary.pdf]

Supplementary material for Electric Field-Induced Settling and Flotation of Flocs in Mixed Aqueous Suspensions of Poly(methyl methacrylate) and Aluminosilicate Hollow Particles

Hiroshi Kimura* and Mirei Sakakibara
Department of Chemistry and Biomolecular Science, Faculty of Engineering, Gifu University, Gifu 501-1193, Japan

* Correspondence: kimura.hiroshi.b1@f.gifu-u.ac.jp; Tel.: +81-58-293-2622

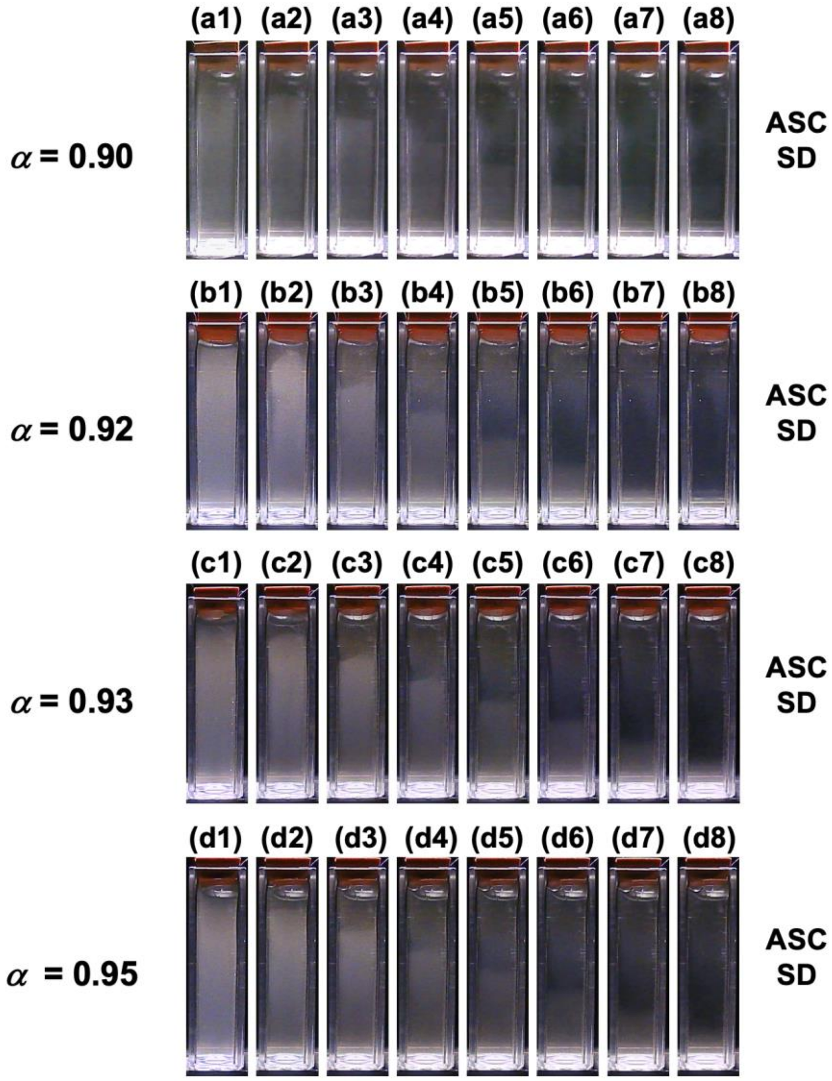

Figure S1. Time-dependent dispersion state of the mixed aqueous suspension of hollow and PMMA particles at 25°C under field-free conditions. (a1–a8) $\alpha = 0.90$, (b1–b8) $\alpha = 0.92$, (c1–c8) $\alpha = 0.93$, (d1–d8) $\alpha = 0.95$. (a1, b1, c1, d1) $t = 0$, (a2, b2, c2, d2) $t = 2000$ s, (a3, b3, c3, d3) $t = 4000$ s, (a4, b4, c4, d4) $t = 6000$ s, (a5, b5, c5, d5) $t = 8000$ s, (a6, b6, c6, d6) $t = 10{,}000$ s, (a7, b7, c7, d7) $t = 12{,}000$ s, (a8, b8, c8, d8) $t = 14{,}000$ s.

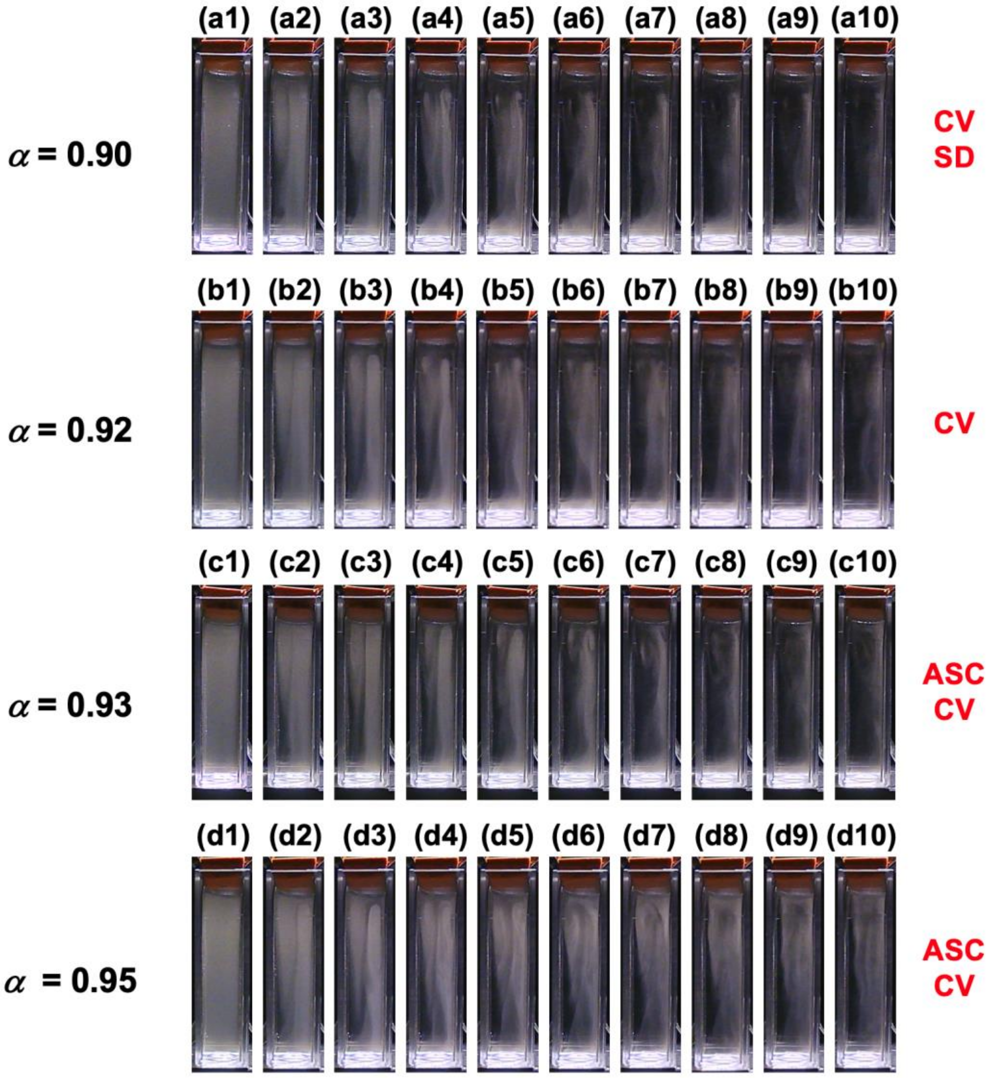

Figure S2. Time-dependent dispersion state of the mixed aqueous suspension of hollow and PMMA particles at 25°C under an applied electric field (E = 0.4 V/mm DC). (a1–a10) α = 0.90, (b1–b10) α = 0.92, (c1–c10) α = 0.93, (d1–d10) α = 0.95. (a1, b1, c1, d1) t = 0, (a2, b2, c2, d2) t = 200 s, (a3, b3, c3, d3) t = 400 s, (a4, b4, c4, d4) t = 600 s, (a5, b5, c5, d5) t = 800 s, (a6, b6, c6, d6) t = 1000 s, (a7, b7, c7, d7) t = 1200 s, (a8, b8, c8, d8) t = 1400 s, (a9, b9, c9, d9) t = 1600 s, (a10, b10, c10, d10) t = 1800 s.
